# Beyond conventional treatment: Novel cell therapies for systemic lupus erythematosus

**DOI:** 10.1016/j.jtauto.2025.100308

**Published:** 2025-08-27

**Authors:** Zeinab Zarei-Behjani, Arghavan Hosseinpouri, Maryam Fotoohi, Akram Shafiee, Dorna Asadi

**Affiliations:** a Department of Tissue Engineering and Applied Cell Sciences, School of Advanced Medical Sciences and Technologies, Shiraz University of Medical Sciences, Shiraz, Iran; b Student Research Committee, Shiraz University of Medical Sciences, Shiraz, Iran; c Department of Pharmaceutics, School of Pharmacy, Hamadan University of Medical Sciences, Hamadan, Iran; d Medicinal Plants and Natural Products Research Center, Hamadan University of Medical Sciences, Hamadan, Iran; e Department of Applied Cell Sciences, Faculty of Medicine, Kashan University of Medical Sciences, Kashan, Iran; f Anatomical Sciences Research Center, Institute for Basic Sciences, Kashan University of Medical Sciences, Kashan, Iran

**Keywords:** Systemic lupus erythematosus, Cell therapy, Stem cell, Exosome

## Abstract

Systemic lupus erythematosus (SLE) is an autoimmune disease characterized by its heterogeneity, as it can affect various organs and exhibit a diverse clinical progression. The identification of SLE relies on the presence of distinct clinical manifestations in the skin, joints, kidneys, and the central nervous system, along with serological markers like antinuclear antibodies such as antibodies targeting dsDNA. The present therapeutic approaches for SLE encompass the use of antimalarial agents, glucocorticoids, immunosuppressive medications, and biological therapies. Despite the advancements in therapeutic strategies, SLE continues to be linked with adverse outcomes. The complicity and unpredictable nature of disease, characterized by episodes of relapses and remissions, coupled with the side effects of current treatment options, the progressive accumulation of organ damage, and persistent mortality rates despite therapeutic improvements, underscores the urgent necessity for the creation of innovative, effective, and specifically targeted therapies. Cell-based therapies, although still in their nascent stages, have attracted considerable interest in the realm of SLE treatment due to their potential for long-term disease suppression or even the possibility of a cure. Various cell types have emerged as promising candidates for SLE management. This review aims to provide a brief overview of the most recent research on novel cell-based therapeutic approaches that have progressed to either pre-clinical or clinical trial phases for the treatment of SLE.

## Highlights

• Systemic lupus erythematosus (SLE) is a heterogeneous autoimmune disease marked by unpredictable flares, multisystem involvement, and limited therapeutic durability.
• Conventional therapies offer only partial disease control and are associated with significant side effects and cumulative organ damage.
• Cell-based therapies offer targeted immunomodulation and potential long-term remission or cure in SLE patients.
• Several cell therapies such as hematopoietic stem cell transplantation (HSCT), mesenchymal stem/stromal cells (MSCs), induced pluripotent stem cells, exosomes, and chimeric antigen receptor-engineered T cells are the most extensively studied and have shown beneficial effects in both SLE pre-clinical and clinical settings.

## Abbreviations

SLE: Systemic lupus erythematosus
ANA: antinuclear antibodies
dsDNA: double-stranded DNA
PBMCs: peripheral blood mononuclear cells
HSCT: Hematopoietic stem cell transplant
GCs: glucocorticoids
CAR: Chimeric Antigen Receptor
MSCs: Mesenchymal Stem cells
iPSCs: induced pluripotent stem cell

## 1 Introduction

Systemic lupus erythematosus (SLE) is an autoimmune disorder marked by its diverse nature, as it can impact multiple organ systems and demonstrate a wide range of clinical symptems. The diagnosis of SLE is based on the observation of specific clinical signs in the skin, joints, kidneys, and central nervous system, in conjunction with serological indicators such as antinuclear antibodies (ANA), particularly those that target double-stranded DNA (dsDNA) [1]. SLE exhibits a remarkable prevalence among females, with nearly 10 female patients for every male affected by condition. T he incidence of the disease varies between 0.3 and 31.5 cases per 100,000 individuals annually and has shown an upward trend over the past four decades, likely attributed to the identification of less severe cases. Globally, adjusted prevalence rates are approaching or surpassing 50–100 cases per 100,000 adults [2]. The incidence, prevalence and mortality of SLE exhibit significant variations across different geographical areas [3]. Various factors, including genetic, environmental, sociodemographic, and methodological considerations, contribute to the observed epidemiological variations. Moreover, these factors affected the unpredictable progression and outcome of the illness [4]. The severity of the disease and the specific organs affected greatly influence the annual direct costs associated with healthcare. It has been estimated that patients with moderate to severe disease in the USA incur costs ranging from at least 3000 to US$12,000, while in Europe, the costs range from 2500 to €5000. These costs reflect the expenses directly related to healthcare services [5]. Despite the progress in therapeutic approach, SLE still remains associated with unfavorable outcomes. The disease's diverse and unpredictable pattern of relapses and remissions, along with the adverse effects of existing treatment regimens, the accumulation of organ damage, and the persistent mortality rates despite therapeutic advancements, highlight the critical need for the development of novel, potent, and specifically targeted medications [6]. Cell-based therapies, despite being in the early stages of development, have garnered significant attention in the field of SLE treatment due to their potential to effectively suppress the disease in the long term or even offer a potential cure. Various types of cells have emerged as promising candidates for the management of SLE [7]. In this review, we present a concise summary of the latest research on novel cell-based therapeutic strategies that have undergone either pre-clinical or clinical trials for the treatment of SLE.

## 2 The pathogenesis of SLE

SLE is an autoimmune disease that affects multiple organs and presents a diverse range of clinical symptoms. These symptoms can vary from mild skin and mucosal manifestations to severe central nervous system involvement, and in some cases, can even lead to mortality [8]. The pathogenesis of SLE includes a complex interaction between the exposome (environmental influence) and genome to produce an epigenetic change that alters the expression of specific genes that contribute to disease development. Several environmental factors such as UVB radiation, infections, and toxins trigger a loss of immune tolerance in genetically susceptible individuals and lead to aberrant activation of autoimmunity [9]. Over the last few years, significant advancements have been made in understanding the genetic characteristics of SLE. This progress has been achieved by consolidating the results from various studies, including genome-wide association studies (GWAS) and whole-exome or whole-genome sequencing studies. In the case of SLE, it is worth noting that only a small portion of cases can be attributed to monogenic disease [10]. It is currently revealed that in SLE patients, a collection of polygenic risk factors, along with an environmental trigger, collaborate to exceed the threshold of disease development [11].

The dysregulation of the immune system in SLE is marked by various abnormalities. These abnormalities affect both the adaptive and innate immunity, providing additional evidence for the complex and multifactorial nature of the underlying mechanisms responsible for the development of the disease [12]. Exposure of self-antigens to the immune cells, possibly from apoptotic cell debris, initiates a feedback loop between innate and adaptive immunity. The production of autoantibodies and immune complexes, the function of autoreactive T cells and B cells, complement activation, and cytokine release cause to extensive tissue damages, manifesting as the clinical feature of SLE [13,14]. T cells play a crucial role in the development of SLE by promoting inflammation through the release of pro-inflammatory cytokines, stimulating B cells to produce autoantibodies, and sustaining the disease through a reservoir of autoreactive memory T cells. However, the composition and function of certain T cell subsets are abnormal in SLE patients [15]. Follicular helper T (Tfh) cells are indispensable for initiating and regulating the germinal center response, facilitating cell proliferation, promoting isotype-switching, and driving somatic hypermutation. Moreover, these cells secrete the cytokine IL-21, which promotes the differentiation of B cells into memory B cells and plasma cells responsible for antibody production [16]. The pathogenesis of SLE is closely linked to abnormal activation of B-cells, resulting in the production of excessive autoantibodies. Dysregulation of several pathways, including the B-cell receptor (BCR), toll-like receptor (TLR), and B-cell activating factor receptor (BAFF-R) pathways, are key factors contributing to this aberrant B-cell activation. These signaling pathways play a multifaceted and interconnected role in the progression and pathogenesis of SLE [17,18]. Following the loss of tolerance in the B cell compartment autoantibodies have emerged in the SLE individuals [19]. Based on their unique characteristics, certain autoantibodies have the ability to determine a specific outcome of the disease. There have been four proposed clusters of autoantigens were identified to involve in SLE pathogenesis: (i) the dsDNA cluster, which is linked to a high occurrence of renal involvement and an increased risk of renal damage; (ii) the Sm/RNP cluster, which is associated with a higher prevalence of pulmonary arterial hypertension and Raynaud's phenomenon; (iii) the anti-cardiolipin and lupus anticoagulant cluster, which is connected to neuropsychiatric involvement, antiphospholipid syndrome, and hemolytic anemia; and (iv) the Ro/La cluster, which does not exhibit any clinical associations [20]. The role of complement system in SLE pathogenesis is attributed to two distinct aspects. Firstly, complement activation results in promoting inflammatory tissue damage. Conversely, genetical deficiencies in complement factors are linked to a heightened susceptibility to developing SLE [21]. On the other side, imbalances in the concentration of cytokines play a role in immune dysfunction, inflammation, and organ damage. The primary cytokine involved in the development of SLE is interferon alpha. The production of interferon alfa is triggered by immune complexes and results in the upregulation of various inflammatory proteins, which contribute to the IFN signature commonly observed in SLE peripheral blood mononuclear cells (PBMCs). Additionally, dysregulation of other cytokines such as IL-6, IFN-γ, IL-17, IL-21, and IL-2 is also observed in SLE [22]. All these factors result in overall dysfunction of the immune system in SLE patients.

## 3 Common treatments for SLE

Current treatment options for SLE include antimalarials, glucocorticoids, immunosuppressants, and biologics [23]. Antimalarials have been utilized for a long time to address SLE, and they continue to hold a significant position in SLE therapy. Their efficacy in managing skin manifestations and arthritis is noteworthy, making them an essential component of the treatment plan for every patient, unless there exists a clear contraindication [24]. This treatment approach not only improves survival rates and reduces disease activity but also offers additional advantages such as cardioprotective and anticancer effects. Moreover, when utilized alongside immunomodulatory treatment, antimalarial therapy has been shown to decrease the need for corticosteroids, prolong the time before reaching end-stage renal disease, and enhance the duration of renal remission [25]. However, retinopathy, gastrointestinal discomfort, and cutaneous reactions even after antimalarials persist to follow with careful consideration [24].

As a first line of treatment, glucocorticoids (GCs) have been utilized for over six decades in the management of SLE [26]. GCs, known for their robust anti-inflammatory and immunosuppressive properties, are highly effective agents with extensive application in the medical field for managing SLE, as well as a range of other conditions including asthma, skin diseases, allergic reactions, and various systemic conditions. GCs exert their effects through various mechanisms including downregulating the production of cytokines and adhesion molecules, thereby reducing inflammation. Additionally, GCs inhibit the migration of immune cells to the site of inflammation and interfere with the functioning of various cell types including leukocytes, fibroblasts, and endothelial cells. These effects are not limited to specific immune cells, as almost all primary and secondary immune cells are susceptible to the actions of GCs. Furthermore, GCs decrease the number of circulating monocytes and macrophages by suppressing their production and releasing from the bone marrow. Moreover, they also decrease the expression of MHC and Fc receptors, as well as the synthesis of pro-inflammatory cytokines such as IL-2, IL-6, TNF-α, and prostaglandins [27]. Nevertheless, the use of glucocorticoids is associated with numerous well-known adverse side effects, such as metabolic disturbances, decreased bone density, and an elevated susceptibility to infections, particularly with prolonged usage [28]. Immunosuppressive agents are frequently employed in combination with glucocorticoids to manage the severe active SLE that affects vital organs. In III and IV stage of lupus nephritis, the primary therapeutic drugs used are cyclophosphamide, mycophenolate mofetil (MMF), and azathioprine [29]. Furthermore, novel immunosuppressants like leflunomide, cyclosporine, and tacrolimus have been gradually integrated into the treatment of SLE, offering additional options for therapy [30]. Encouragingly, in January 2021, the FDA approved voclosporin, an oral calcineurin inhibitor immunosuppressant, in combination with MMF for adults with active lupus nephritis. Nevertheless, it is important to note that immunosuppressants have a broad spectrum of effects and lack precise targeting, which can result in side effects such as infection and organ damage [26]. Belimumab and anifrolumab are the two biologics that have been approved for the treatment of patients diagnosed with SLE. While rituximab (RTX) is not officially approved for SLE treatment, it is sometimes prescribed off-label. Additionally, numerous other biological drugs are currently being assessed in SLE clinical trials. It is advisable to contemplate the use of biologics in cases of persistently active or recurrent SLE [31]. Bispecific antibodies which are recombinant monoclonal antibodies engineered to bind two different antigens or epitopes via dual antigen-binding domains, cause more potent results [32]. In SLE, bispecific antibodies specifically applied for simultaneously targeting distinct immune cell populations (B and T cells), Blocking two cytokines involved in B cell survival and inflammation, modulating immune checkpoints to restore self-tolerance [33,34]. Several clinical preclinical studies have demonstrated proof-of-concept for bispecific antibody therapy in lupus. After dual targeting of BAFF and ICOSL by AMG 570, the enhancement of B cell depletion and improvement of renal histology was determined in lupus models of mice and monkeys [35]. Mosunetuzumab is a humanized IgG1 monoclonal bispecific antibody that targets both CD20 and CD3, thereby engaging T cells to promote the elimination of B cells through the facilitation of antibody-dependent cellular cytotoxicity (ADCC). Currently, a clinical trial is in progress to assess the safety, tolerability, pharmacokinetics, and pharmacodynamics of mosunetuzumab administered subcutaneously to individuals diagnosed with SLE [34]. Additionally, CLN-978 whit bispecific structure targets CD19 and CD3 to engage T lymphocytes in targeting autoimmune B lymphocytes. A phase 1 clinical trial was conducted to assess the safety, pharmacokinetics, and immunogenicity of CLN-978 for treatment of moderate to severe SLE after FDA allowance.

**Fig. 1:**
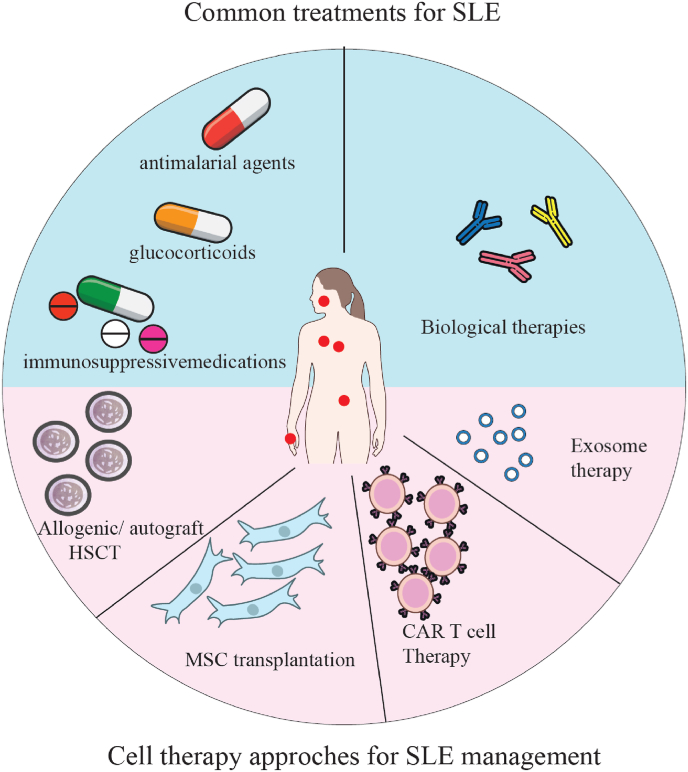
Common and cell-based therapy approaches for management of SLE. Current treatment of SLE including antimalarials, glucocorticoids, immunosuppressants, and biologics exhibit the prevention of SLE relapses in more than 50 % of treated patients with several side effects. There is an increasing interest in investigating alternative treatment methods, including cell therapies like HSCT, MSC transplantation, CAR T cell therapy and cell free agents.

Recently, new technologies have been developed for management of SLE, in which cell therapies are described in this review. Fig. 1 illustrates both the common and alternative therapies for SLE management (Fig. 1).

## 4 Cell therapy approach for SLE management

**Fig. 2:**
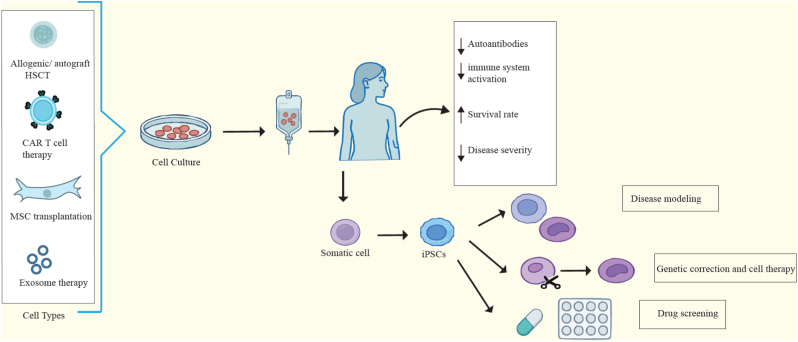
Cell therapy options for management of SLE. Different types of cell therapies are explored for treatment of SLE patients like HSCs, MSCs, iPSCs, CAR T cell therapy and cell-free approaches.

Despite the advancements in understanding the pathogenesis of the disease, current immunosuppressive therapies have not been successful in preventing SLE relapses in more than 50 % of treated patients. As a result, there is growing interest in exploring alternative treatment options such as cell therapy (Fig. 1). This approach aims to address the limitations of traditional treatments and improve the recovery rate for patients with refractory SLE. Over the years, various types of cells, including hematopoietic stem cells (HSCs) and mesenchymal stem cells (MSCs) induced pluripotent stem cells (induced pluripotent stem cell (iPSC)), Chimeric Antigen Receptor T cell therapy and cell-free approaches have been utilized in this field (Fig. 2). Here, we provide an overview of different sources of cells and their applications in the treatment of SLE (Table 1).

**Table 1:** Cell therapy approaches for SLE treatment.

Cell Therapy	Source	Mechanism of Action	Clinical Stage	Key Advantages	Key Limitations
Hematopoietic Stem Cell Transplantation (HSCT)	Bone marrow, peripheral blood, umbilical cord	Immune system reset via myeloablation and reconstitution	Clinical trials (Phase I/II)	Potential long-term remission	High risk of infection, relapse, and treatment-related mortality
Mesenchymal Stem Cells (MSCs)	Bone marrow, adipose tissue, umbilical cord	Immunomodulation via cytokine secretion and T/B cell regulation	Preclinical & Clinical (Phase I/II)	Low immunogenicity promotes immune tolerance	Functional impairment in autologous MSCs, inconsistent efficacy
CAR T Cells	Autologous T cells engineered to express CARs	Targeted depletion of autoreactive B cells via CD19	Early clinical trials	High specificity, durable responses in refractory SLE	Risk of cytokine release syndrome, long-term safety unknown
iPSCs	Reprogrammed somatic cells	Disease modeling, drug screening, personalized therapy	Preclinical	Patient-specific applications, genetic modeling	Ethical concerns, tumorigenicity, limited therapeutic use
MSC-derived Exosomes	Secreted vesicles from MSCs	Anti-inflammatory signaling, immune cell modulation	Preclinical	Cell-free, low immunogenicity, good stability	Challenges in production, heterogeneity, targeting specificity

## 5 Hematopoietic stem cell transplantation

HSCs are undifferentiated multipotent cells with the ability to differentiate into various types of blood cells, encompassing both myeloid and lymphoid cells. Under the normal condition, a rare population of HSCs which is called long-term repopulating HSCs remains in a quiescent state within the bone marrow (BM) niche to maintain their long-term self-renewal potential and prevent depletion of stem cells [36]. LT-HSCs are responsible for generating short-term repopulating HSCs (ST-HSCs) and multipotent progenitors (MPPs). These cells exhibit a wide range of differentiation capabilities and undergo rapid proliferation, however, ST-HSC and MPP are lacking the ability to self-renew over the LT-HSCs. Subsequent stages of differentiation give rise to lineage-specific progenitors, ultimately leading to the production of fully mature cells [37]. HSCs have the ability to be mobilized from the bone marrow niche and enter peripheral blood with the aid of hematopoietic cytokines. This process enables a convenient collection of these cells for clinical transplantation purposes [38]. Umbilical cord blood, obtained from the placenta and cord after childbirth, serves as a valuable reservoir of HSCs and offers an alternative to bone marrow transplantation. The HSCs found in cord blood possess a more primitive nature compared to those in bone marrow or peripheral blood, and they exhibit numerous advantages, notably their remarkable capacity for proliferation [39]. Hence, these multipotent cells can be isolated for clinical use from multiple sources, including peripheral blood (PB), bone marrow (BM), and umbilical cord blood (UCB).

Hematopoietic stem cell transplant (HSCT), also known as bone marrow transplant, is a medical procedure where healthy HSCs are infused into individuals with compromised or insufficient bone marrow. This therapeutic intervention offers numerous advantages. Firstly, it aids in enhancing the functionality of the bone marrow. Furthermore, depending on the specific ailment being addressed, it has the potential to eradicate cancerous tumor cells. Moreover, it facilitates the production of fully functional cells that can replace malfunctioning ones [40]. Autologous HSCT involves using the patient's own harvested HSC, which are reinfused after myeloablation. On the other hand, allogeneic HSCT utilizes human leukocyte antigen (HLA)-matched stem cells obtained from a donor. Every year, more than 25,000 HSCTs are conducted worldwide to treat various medical conditions such as lymphoma, leukemia, immune-deficiency illnesses, congenital metabolic defects, hemoglobinopathies, and myelodysplastic and myeloproliferative syndromes [41].

In the past 25 years, there has been a growing utilization of HSCT as a therapeutic approach for individuals suffering from severe and chronic autoimmune diseases. The underlying principle behind employing HSCT in the treatment of autoimmune diseases is to essentially “reset” the immune system by eliminating the existing dysfunctional immune cells and replacing them with a fresh and robust population of immune cells [42,43]. Autologous HSCT has emerged as a potential alternative treatment for individuals with SLE who do not respond to conventional therapies [44]. This therapeutic approach holds promise in arresting the progression of the autoimmune disease and managing long-term remission in refractory cases. The effectiveness of autologous HSCT in severe lupus is believed to arise from its ability to deplete autoreactive immunologic memory and reset the adaptive immune system [45]. Several clinical trials were conducted to assess the therapeutic effects of HSCT for SLE patients that is summarized in Table 2. The majority of studies indicated that remission was achieved in most patients. However, the incidence of the relapse of the original disease rose with long-term follow-up periods. Frequently reported adverse effects included infections and secondary autoimmune disorders. The assessments of these clinical trials revealed some limitations that hindered the generalizability of the study findings. The very effective challenges are short follow-up duration and the absence of randomized controlled trials. While HSCT presents a feasible option for SLE patients, its precise clinical effectiveness requires further assessment through precisely designed studies [44]. Altogether, HSCT has been associated with notable rates of mortality and morbidity, alongside a substantial risk of relapse and opportunistic infections. This phenomenon may be linked to the deficiencies present in bone marrow stem cells and the abnormal immune responses characteristic of patients suffering from SLE. Research has further revealed that both genetic patterns and inflammatory processes play a critical role in determining the quantity and functionality of HSCs in a lupus murine model. As a result, the management of SLE remains a complex and challenging endeavor [46].

**Table 2:** The therapeutic effects of HSCT for SLE patients.

Type of intervention	Number of patients	Patient's characteristics	Main outcomes	Country	Ref
Autologous HSCT Vs. immunesupression and assessed outcome in pregnancy after remission	11	HSCT-11, SLEDAI^a^ 20 ± 5, nontransplant—39, SLEDAI 21 ± 3	HSCT-SLEDAI preconception 4 ± 1, postpartum 4 ± 1,no lupus nephritis/hypertension duringpregnancy, non-transplant SLEDAI preconception 4 ± 2, postpartum 7 ± 2, 33 % hypertension and31 % lupus nephritis duringpregnancy.	China	[47]
Intervention autologous HSCT, Control conventional therapy (Steroids and cyclophosphamide (CYC)	Intervention—17 patients, Control—20 patients	Transfusion dependent autoimmune cytopenias, severe pericarditis, involvement of the lung and central nervous system, glomerulonephritis.	Intervention—16/17 steroidsstopped within one year. SLEDAI in 5 years 32.3 ± 9.2 to 0.76 ± 0.92 (p < 0.01), Control—15/20 achieved remission, SLEDAI in 5 years 18.21 ± 5.71 to6.28 ± 4.48 (p < 0.01)	China	[48]
Autologous. Unmanipulatedstem cells in 18. Conditioning regimelow, moderate intensity	28	Patients with SLE	Median follow up 38 months. Five-year overall survival 81 ± 8 %, relapse incidence 56 ± 11 %, disease free survival 29 ± 9 %	Europe	[49]
Autologous. Conditioning CYC with anti-thymocyte globulin (ATG)/anti-lymphocyte globulin (ALG)/total body irradiation (TBI)	27	Severe SLE	6 months—two partial remission, 21 remission, 10 years-one remained active, four lost to follow up, 16 remained in remission, 14 with lupus nephritis 4 g/24 h pretreatment to 0 g/24 h at 5 and 10 years.	China	[50]
Autologous peripheral blood HSCT	22	Patients with lupus nephritis and failed previous therapy orother significant organ involvement	10 patients relapsed within 10 years of median follow up. Five year progressionfree survival was 67.9 %	China	[51]
Priming with rituximab, MPP and CYC. Conditioning with fludarabine, rituximab and CYC	8	Patients with severe, active lupus refractory to immunesuppressionbetween 15 and 40 years	In follow-up up to 3 years two patients havemaintained complete remission with SLEDAI 0	USA	[44]
dose-intense immune suppression and autologous hematopoietic stem-cell (CD34)	7	Patients with SLE experienced persistent multiorgan dysfunction, despite standard doses of intravenous CYC	At a median follow-up of 25 months [[12], [13], [14], [15], [16], [17], [18], [19], [20], [21], [22], [23], [24], [25], [26], [27], [28], [29], [30], [31], [32], [33], [34], [35], [36], [37], [38], [39], [40]], all patients were free from signs of active lupus. Renal, cardiac, pulmonary, and serological markers, and T-cell phenotype and repertoire had normalized.	USA	[52]
Autologous peripheral blood and BM HSCT	53	Severe SLE	Cumulative patient survival was 84 % at 12 months and 62 % at 48 months. (66 %) had remission of lupus activity (SLEDAI <3). Remission was not associated with any pre-autologous stem cell transplantation (ASCT).	Europe	[53]
Autologous peripheral blood HSCT	50	severe and treatment-refractory SLE	Treatment-related mortality was 2 % (1/50). With a mean follow-up of 29 months (range, 6 months to 7.5 years) for patients undergoing HSCT, overall 5-year survival was 84 %, and probability of disease-free survival at 5 years following HSCT was 50 %.	USA	[54]
Concurrent administration of CYC, MMP, fludarabine and rituximab and Autologous peripheral blood HSCT.	8	Patients with SLE refractory to treatment	Five of eight patients achieved a complete response, including a decline in the SLEDAI to zero, which was sustained in four patients for a median of 165 months. One patient achieved a partial response. Two patients with nephritis and underlying comorbidities in most organs had early deaths from infection and multiorgan failure.	USA	[55]
Autologous peripheral blood HSCT	7	Patients with refractory systemic lupus erythematosus (SLE)	Depletion of autoreactive immunologic memory, reflected by the disappearance of pathogenic anti-double-stranded DNA (dsDNA) antibodies and protective antibodies in serum and a fundamental resetting of the adaptive immune system. regeneration of thymic-derived FoxP3(+) regulatory T cells, and normalization of peripheral T-cell receptor. Responders exhibited normalization of the previously disturbed B-cell homeostasis with numeric recovery of the naive B-cell compartment within 1 year after ASCT.	Germany	[45]
Allogenic HSCT with conditioning regimen included thymoglobulin busulfan, thiotepa, fludarabine, rituximab, CYC, MMF, tacrolimus	1	The patient did not respond to rigorous immune suppression treatment with methylprednisone pulse therapy plus MMF and intravenous immunoglobulin infusions as well as the citrulline supplementation for Lysinuric protein intolerance (LPI).	The patient's fever was controlled and all hemophagocytic lymphohistiocystosis-related markers returned to their normal ranges. the patient's hepatosplenomegaly improved. Autoantibodies, including antinuclear antibodies and anti–double-stranded DNA, were negative, and the patient's erythrocyte sedimentation rate returned to normal.	China	[56]
Autologous peripheral blood HSCT	20	Severe SLE	– Recruiting	USA	NCT05029336

a Systemic Lupus Erythematosus Disease Activity Index.

## 6 Mesenchymal stem cell therapy

Mesenchymal stem cells (MSCs) possess the unique ability to both self-renew and differentiate into a diverse array of cell types. These multipotent stem cells exhibit the ability to differentiate into various types of mesodermal tissues, such as osteocytes, chondrocytes, and adipocytes. Moreover, under suitable developmental settings, they can also differentiate into non-mesodermal lineages like hepatocytes, neurons, pancreatic cells, cardiac muscle cells, or astrocytes [57]. The International Society for Cellular Therapy has recommended a set of minimum criteria to harmonize the characterization of MSCs worldwide. These cells [1] must demonstrate plastic adherence [2] should have a specific combination of cell surface markers such as CD73, CD90, CD105, and should not express CD14, CD34, CD45, and human leukocyte antigen-DR (HLA-DR), and [3] must possess the capability to differentiate into adipocytes, chondrocytes, and osteoblasts *in vitro* [58].There exist numerous sources of human derived MSCs utilized in regenerative medicine. These sources can be categorized into two types: adult MSCs, which isolated from bone marrow, adipose tissue, peripheral blood, and dental pulp, and neonatal tissue-derived MSCs, acquired from placenta, amnion, and umbilical cord [59].

MSCs possess immunosuppressive characteristics that present a promising therapeutic approach for the treatment of autoimmune diseases [60]. *In vitro* studies demonstrate that MSCs can inhibit nearly all forms of immune responses and can prevent disease onset in various experimental models of autoimmunity. However, the mechanisms underlying the pathogenesis of human autoimmune diseases are significantly more complex, and it is crucial to note that treatment cannot be initiated prior to the onset of the disease. In cases of autoimmune disorders, ongoing antigenic stimulation leads to the recruitment of endogenous MSCs to the affected areas, which may contribute to the development of fibrosis [61]. Several studies investigating the immunomodulatory effects of MSCs in preclinical models of SLE have demonstrated their beneficial properties. In SLE animal models, MSCs exhibited immunosuppressive functions through the secretion of proinflammatory cytokines and the inhibition of lymphocyte activation and proliferation. The link between SLE and the activation and proliferation of autoreactive B cells, as well as certain T cell subtypes, is well-established. Additionally, a deficiency in both anti-inflammatory (Treg, Th2) and proinflammatory (Th17, Th1) subsets is acknowledged as a critical factor in the pathogenesis of SLE, leading to tissue inflammation, immune dysregulation, and the risk of multiorgan failure [62]. Then, the administration of MSCs may recover the unbalanced condition of the immune system in SLE model.

The differed symptoms of lupus in humans present considerable obstacles, as mouse models often do not adequately reflect the comprehensive spectrum of pathophysiological alterations observed in the human population [63]. A comprehensive analysis regarding safety, effectiveness, and signaling mechanisms linked to stem cell therapy for SLE revealed significant potential for clinical use but more long-term studies with larger number of patients need to be conducted to demonstrate the outcomes [64]. The required MSCs for transplantation in SLE patients are derived from both autologous or allogeneic sources. The primary obstacle to the clinical implementation of autologous MSC therapy in patients with SLE was identified as functional impairments, which included alterations in the cytoskeleton, abnormal cytokine production, compromised cellular phenotype, reduced proliferation, and defective hematopoiesis. As a result of these dysfunctions, allogeneic MSCs were favored for transplantation in SLE patients. Clinical investigations involving allogeneic MSCs sourced from bone marrow and umbilical cord demonstrated a capacity to mitigate autoimmunity and restore renal function in affected individuals. This therapeutic effect was achieved by reestablishing a balance between Th1 and Th2 cytokines, reducing levels of IL-4, and enhancing the populations of regulatory T cells, TGF-β, and IFN-γ [65,66]. Table 3 summarizes some *in vitro*, preclinical and clinical studies that are performed to target SLE with MSC.

**Table 3:** *In vitro*, preclinical and clinical studies in the field of MSC therapy for targeting SLE.

Type of study		The characteristics of studies				
	Type of MSC	MSC counts	Type of target cells	Main outcomes		References
*In vitro*	Human UC-MSC	2 × 10^4^	Macrophages	The results showed that the nucleotide-binding oligomerization domain-like receptor 3 (NLRP3) inflammasome was activated in macrophages from MRL/lpr mice and patients with SLE, correlating with disease activity.		[67]
	Modified mesenchymal stem cells	1 × 10^4^ cells per well	Splenocyte	These cells had enhanced immunosuppression *in vitro* in terms of inhibiting splenocyte proliferation, reducing proinflammatory factors (IL-1*β*, TNF-*α*, IL-17, and IL-6), and suppressing autoantibodies (anti-dsDNA and anti-ANA).		[68]
	Human UC-MSCs	the T lymphocytes were transferred to UC-MSC culture wells at a cell number ratio of 5:1	T lymphocytes	C-MSCs exert significant regulatory effects on cellular and humoral immunity in the T lymphocytes of SLE patients. UC-MSCs have shown a potential therapeutic pathway in SLE by down-regulating OPN, TNF-a and NF-kB and up-regulating. miR-181a in T lymphocytes.		[69]
	Human adipose tissue derived MSCs	1 × 10^5^	Macrophage cells	The IL-10 levels of all groups were significantly higher compared to the unstimulated macrophage -PBMC group. MSCs were found to differentiate macrophages into a distinctive phenotype, which was close to the M2c phenotype, but was not considered as an M2c cell due to the low expression of CD163, a characteristic marker for M2c. Treg cell activation caused by direct interactions between MSCs and macrophage cells may be the most prominent observation		[70]
	Type of MSC	MSC counts	Type of animals	Route of administration	Main outcomes	References

preclinical	Human adipose derived- MSC	1 × 10^6^ ASCs/150 μL	MRL/lpr mice	Intravenous	ASC transplantation led to a significant improvement in hyperkeratosis and inflammatory cell infiltration scores in histopathological sections. MRL/lpr mice treated with ASC had a significant reduction in serum levels of IL-17 and IL-4, as well as a reduction in the level of IFN-γ and the ratio of Th1/Th2 (IFN-γ/IL-4) compared to the saline-treated control group.	[71]
	Bone mesenchymal stem cells	2 × 10^6^ cells/mouse	MRL/MpJ-Faslpr mice	via ip injections	BMSCs significantly promoted the expression of IL-10 while reducing IL-18. Moreover, BMSCs exert immunomodulatory effects by reducing Th17 expression and rectifying the Th17/Treg imbalance.	[72]
	Human UC-MSCs		Lupus-prone mice		Proteinuria levels decreased in the UC-MSCs-treated group and were maintained over time, but the IL-2 group returned to the pre-treatment level 4 weeks later. UC-MSCs treatment provided long-term alleviation of renal damage. UC-MSCs treatment maintained long-term inhibition of immune complexes deposition.	[73]
	Human MSCs!!! منبع سلول گفته نشده	1 × 10^6^ cells/mL	MRL/*lpr* mice	Intraperitoneal	A significant reduction of TNF-α, IL-6 and TGF-β could be observed in MSCs compared with controls. indicating a relatively lower in-flammatory level. erum creatinine and blood urea nitrogen reflected glomerular filtration which was distinct from glomeruli inflammatory.	[74]
	57/B6 mice bone marrow MSCs (BM-MSCs)	5 × 10^5^ BM-MSCs/0.2 ml of PBS	B6.MRL-*Fas*^*lpr*^ mice	Injected via tail vein	Eight weeks post BM-MSCs transplantation, the concentrations of Qa-2 (Qa-2 is the functional homolog of HLA-G in mice) in BM-MSCs transplanted mice were significantly higher than that in mice receiving no therapy and normal controls.	[75]
	Human UC-MSC	5 × 10^5^	MRL/lpr mice	via the tail vein	After MSC transplantation, the disease severity in MRL/lpr mice was alleviated, and NLRP3 inflammasome activation was inhibited with decreased levels of NLRP3 and caspase-1 in macrophages.	[67]
	Modified mesenchymal stem cells	1 × 10^6^ cells/200 ml PBS	MRL/*lpr* mice (model of SLE)	Injected via tail vein	Improved survival and reduced signs of SLE compared with controls. Expression of IL-37 by mesenchymal stem cells can maintain higher serum levels of IL-37, and these cells had prolonged survival after transplantation, perhaps through IL-37 suppressing the inflammatory microenvironment.	[68]
	Type of MSC	Allogeneic/autograft	Number and characteristics of patients	Route of administration	Main outcomes	References

Clinical trial	Human UC-MSC	Allogenic	10 patients with active SLE, refractory to conventional therapies	MSCs were slowly infused by a heparinized syringe through the cubital vein of the arm over 30 min.	The expression of ILT2 on CD4 + T cells from SLE patients decreased significantly compared to that of healthy controls. A positive correlation between the frequencies of Treg and CD4 + ILT2 + T cells was found in SLE patients. The levels of sHLA-G increased 24 h post UC-MSCs transplantation.	[75]
	BM-MSC were obtained from healthy members under 30 years of patients' Families (8 patients). UC-MSC (27patients)	Allogenic	Thirty-five SLE patients with refractory cytopenia	Infused	The result suggested that MSCT could reverse hematological aberration in SLE patients with refractory cytopenia, which might be associated with reconstitution of Treg and Th17	[76]
	UC –MSC	Allogeneic	40 patients with active and refractory SLE.	Infused intravenously	A significant decline in SLEDAI and BILAG scores as well as proteinuria, serum creatinine, and urea nitrogen. Additionally, serum concentration of albumin and complement amplified after UC-MSC infusion.	[77]
	UC –MSC	Allogeneic	11 SLE patients refractory to conven-tional therapies	1 million cells per kilogram of body weight were administered by intravenous infusion	lower serum levels of interleukin (IL)-1β and IL-18 were observed in patients with SLE who underwent MSC transplantation. MSC ameliorates SLE by preventing NLRP3 inflammasome activation by Pim-1. the expression of NLRP3 and the pro-inflammatory molecules in the serum were significantly decreased after MSC transplantation.	[67]
	UC-MSC	Allogeneic	Twenty-one SLE patients refractory to conven-tional therapies	1 million cells per kilogram of body weight were administered by intravenous infusion	Interferon-γ induces FLT3L expression in UC-MSCs through JAK/STAT signaling pathway. Thus, allogeneic MSCs might suppress inflammation in lupus through up-regulating tolerogenic DCs. the number of CD1c + DCs in PBMCs in SLE patients decreased compared with healthy controls and RA Patients. The level of FLT3L significantly decreased in peripheral blood in patients with SLE. Allogeneic UC-MSCs show unique immunoregulatory func-tions by suppressing T cell proliferation and inducing the gen-eration of Treg, inhibiting B cell function and IgG production, or promoting the phagocytic activity of macrophage.	[78]
	UC-MSC	Allogenic	Thirteen patients	1 × 10^6^/kg of body weight administered by intravenous infusion.	Clearance of Apoptotic Cells(ACs) by MSCs contributes to immunosuppressive function via increasing PGE2 production. These findings reveal a previously unrecognized role of MSC-mediated phagocytosis of ACs in MSC-based immunotherapy.	[79]
	AD-MSC	Allogeneic	nine biopsy-proven LN (lupus nephritis) patients refractory	systemic infusion of 2 × 10^6^ allogeneic adipose-derived (AD) MSCs/kg	Allogenic AD-MSC transplantation was associated with favourable safety and efficient to reduce urine protein excretion and disease activity. Median score of Systemic Lupus Erythematosus Disease Activity Index decreased significantly from 16 at the baseline to 6 at sixth months post-treatment.	[80]
	Pooled allogenic mesenchymal stem cell derived from olfactory mucosa	Allogenic	10 patients with active lupus nephritis	–	Completed	NCT04184258
	UC-MSC	Allogenic	6 Clinically mild to moderately active SLE determined by SLEDAI score ≥4 and ≤ 10 at screening	1 × 10^6^ cells/kg	glycoprotein A repetition predominant (GARP)- -TGFβ complexes were significantly increased following the MSC infusions. The B cell changes and the GARP-TGFβ increases significantly correlated with changes in SLEDAI scores.	[81] NCT03171194
	BM-MSC	Allogenic	10 patients with lupus nephritis and lupus cytopenia	2.0 × 10^6^ cell/kg intravenous infusions	Recruiting	NCT04835883
	UC-MSC	Allogenic	81	1 × 10^6^ cells/kg intravenous infusions	Recruiting	NCT02633163

Despite the remarkable results in MSC therapy for SLE treatment, it is important to note that the administration of MSCs in the context of chronic inflammatory conditions may not be absolutely advantageous. Several literatures have emphasized that MSCs are not intrinsically immunosuppressive; rather, they require a 'licensing’ process facilitated by acute phase inflammatory molecules such as IFNγ and TNF-α, or by ligands that activate toll-like receptors (TLRs). In contrast, exposure to certain cytokines or the activation of specific TLRs can induce MSCs to adopt an immunostimulatory role [82]. Therefore, a thorough examination of the inflammatory situation/present in autoimmune diseases is essential to determine the optimal conditions for effective MSC therapy.

## 7 Pluripotent stem cells

Induced pluripotent stem cells (iPSCs) are embryonic-like cells produced by introducing specific genes into somatic cells. This induced pluripotency enables them, like embryonic cells, to have potential of differentiation into different types of cells. They are able to remodel and retrieve their silent pluripotent capabilities [83,84]. Today, iPSCs are introduced as an important tool for modeling and treating SLE (Table 4). For the investigation of cellular and molecular mechanisms underlying specific diseases such as SLE, iPSCs are reprogrammed from patient-specific somatic cells because they can carry disease-related mutations in their genome. These cells can be differentiated into a variety of cell types, rendering them very useful in studies on the pathogenesis of SLE [85]. For instance, iPSCs have been derived from SLE patients and then differentiated into relevant immune cell types, including B and T cells. Such patient-specific iPSCs have been used to model abnormalities in immune cell function and signaling pathways that lead to disease, providing insights into the dysregulated immune responses observed in SLE [86,87]. Indeed, such iPSCs have facilitated the study of genetic factors associated with SLE. These capabilities have included modeling how those genetic factors contribute to disease development and progression by generating iPSCs from patients carrying specific genetic variants linked to SLE, including polymorphisms in genes encoding components of the type I interferon pathway. This has enabled the unraveling of complicated interactions of genetic predisposition with environmental triggers in SLE that constitute potential targets for the diagnosis and medical intervention of SLE [88,89]. Besides the applications in disease modeling, iPSCs hold immense promises for drug discovery and personalized medicine for SLE patients. High-throughput drug screening using iPSC-derived cells, such as renal cells commonly targeted in lupus nephritis, identifies novel therapeutic agents with the capability to reduce disease symptoms or prevent organ damages. This approach allows for drug testing in a patient-specific setting, highly relevant in SLE owing to variable treatment responses among the subjects [90]. Furthermore, the use of iPSCs in individualized medicine is promising for tailoring treatments to personal SLE patients based on their unique cellular and genetic profiles [87]. By generating iPSCs from SLE patients and differentiating them into the affected cell types, researchers can evaluate the effectiveness and toxicity of different therapies on a case-by-case basis, minimizing adverse effects and maximizing therapeutic efficacy. This approach could lead to more precise and effective treatment strategies, ultimately improving outcomes for patients with SLE [91].

**Table 4:** Induced pluripotent stem cells (iPSCs) applications in SLE.

Aspect	Details
Definition	iPSCs are somatic cells reprogrammed to an embryonic-like pluripotent state via gene induction.
Differentiation Potential	Can differentiate into various cell types, including B and T lymphocytes.
Disease Modeling	iPSCs from SLE patients carry disease-related mutations, enabling modeling of immune dysfunction and signaling abnormalities.
Genetic Studies	iPSCs are used to study polymorphisms (e.g., type I interferon pathway genes) and their role in SLE pathogenesis.
Drug Discovery	Used in high-throughput screening with patient-specific iPSC-derived cells (e.g., renal cells) to identify new therapeutic compounds.
Personalized Medicine	Enable testing of drug efficacy and toxicity in a patient-specific context, allowing individualized treatment optimization.
Therapeutic Potential	Hold promises to develop precision therapies to improve efficacy and reduce side effects in SLE patients.

## 8 Chimeric antigen receptor T cell therapy

CAR T cell therapy is an emerging immunotherapy that has been used to treat patients with relapsed or resistant cancers in numerous clinical trials [92,93]. In CAR T-cell therapy, autologous T cells are collected through leukopheresis and subjected to genetic modification. The modified T cells are then expanded in culture and prepared CAR T-cell products undergo quality control tests. Before the infusion, to increase the growth and durability of CAR T cells inside the body, patients undergo chemotherapy [94,95]. CARs are composed of two main components: extracellular domain and intracellular domain. The extracellular domain consists of variable parts of the light and heavy chains of specific antibodies (single-chain variable fragments) that are connected to each other and play a critical role in target recognition. The intracellular part of CAR consists of one or more signaling domains according to the pathway that mediates signal transduction specific to T cell receptor (TCR) after binding to the target. These two parts are connected to each other by a linker peptide or spacer [96,97]. The primary benefit of CARs lies in their ability to recognize antigens The primary benefit of CARs lies in their ability to recognize not only antigens presented within the context of MHC class I or II but also to identify entire antigens, encompassing both complete protein and non-protein targets. The main advantage of CARs is that they are not limited to the recognition of antigens presented in the MHC class I or I context, holding the capacity to recognize the entire antigens, including complete protein and non-protein targets [98]. Another notable advantage of CAR technology is its “off-the-shelf” applicability in a translational setting, unlike T-cell receptors that are restricted by the major histocompatibility complex (MHC) [99].

CAR-T cell therapy was originally developed to treat cancer; however, this approach has recently been used for autoimmune diseases including SLE. Production of sufficient amounts of functional autologous CAR T cells from SLE patients has been possible. High expansion and survival rates of CAR T cells were observed in SLE patients, which resulted in CAR T cells in sufficient dose and quality for clinical use. CAR T cells from every patient exhibited distinct *in vitro* cytotoxity against CD 19 cell lines [100]. In a study, anti-CD19 CAR-T cell therapy was used to treat SLE in a mouse disease model. They constructed murine anti-CD19 CARs with CD28 or 4-1BB as an intracellular stimulatory motif, and by injecting them into MRL-lpr mice, the therapeutic function of the corresponding CAR-T cells was evaluated. Additionally, anti-CD19 CAR-T cells were transferred into MRL-lpr mice before disease onset to determine their role in preventing SLE. The anti-CD19 CAR-T cells transplation not only stopped the progression of the disease before symptoms appeared, but it also showed therapeutic advantages before or after the progressing disease [101]. CAR T cells targeting CD19 inhibited the production of autoantibodies, ameliorated disease symptoms in affected organs, and significantly prolonged survival beyond typical lifespans in the (NZB × NZW) F1 and MRL fas/fas murine models of lupus. These CAR T cells exhibited persistent *in vivo* activity for up to one year and were predominantly found within the CD44^+^CD62L + T cell subset. Furthermore, splenic T cells obtained from mice treated with CAR T cells effectively depleted CD19^+^ B cells and mitigated disease in naive autoimmune mice, suggesting that the control of the disease was mediated by cellular mechanisms [102].

In a limited set of human trials involving CD19-CAR T cell therapy for SLE, patients who treated with their own engineered T cells showed considerable growth of CAR T cells in their body, rapid improvement in lupus symptoms, and minimal or no side effects [103]. A 20-year-old woman with severe SLE resistant to numerous therapies with active lupus nephritis, nephrotic syndrome, pericarditis, pleuritis, rash, arthritis was treated with CD19-targeted CAR-T cell therapy. The level of dsDNA autoantibodies quickly decreased, and the low levels of C3 and C4 were normalized after receiving CAR T cell therapy. Furthermore, the patient did not exhibit any adverse side effects, such as neurotoxicity, cytokine release syndrome or prolonged cytopenia. These findings suggest that CAR T cell therapy could remission of refractory SLE [104]. The adoptive transfer of CD19-targeted CAR T cells led to a significant reconfiguration of B cells in a limited case series involving eight patients suffering from treatment-refractory SLE. This intervention resulted in the elimination of autoreactive antibodies and sustained remission, including the resolution of lupus nephritis, with follow-up periods ranging from 6 to 29 months [105]. The treatment of a 15-year-old girl suffering from SLE as the first pediatric patient with anti-CD19 CAR T cells resulted in sustained remission of a rapidly progressing and refractory form of lupus nephritis. Following the administration of CAR T cells, there was a swift reduction in the activity of disease. Symptoms associated with arthritis resolved and complement levels of C3 and C4 returned to normal within six weeks. Additionally, anti-nuclear antibodies, including anti-dsDNA, as well as all other autoantibodies, were no longer detectable. Renal function showed significant improvement, allowing for an extension of haemodialysis intervals from one-week post-CAR T cell infusion, with the final haemodialysis session occurring on day 17. Mild cytokine release syndrome was initially managed with antipyretics, and the patient was discharged from the hospital on day 11 without any indications of prolonged bone marrow toxicity or immune cell-associated neurotoxicity syndrome [106].

## 9 Extracellular vesicles

Extracellular vesicles (EVs) represent a heterogeneous class of membrane-bound structures derived from different types of cells. These vesicles include apoptotic bodies, microvesicles, and exosomes, which are different in size, composition, and mechanism of biogenesis [107]. EVs encapsulate a diverse array of cytoplasmic and membrane components, that are selectively incorporated during the vesicle biogenesis process [108]. Exosomes are enriched in a variety of biomolecules, including proteins, transcription factors, lipids, DNA, microRNAs (miRNAs), and messenger RNAs (mRNAs). They exert their effects on target cells either by binding to specific receptors located in target tissues or through fusion with the plasma membranes of recipient cells [109]. The exosomal membrane is characterized by enrichment of transmembrane markers such as CD9, CD63, CD81, and TSG101, along with RAB family proteins, which are involved in vesicle trafficking and signal transduction [110]. Exosomes are regarded as optimal delivery vehicles due to their stability, minimal toxicity, low immunogenicity, and specific targeting capabilities [111]. Recent studies have identified exosomes as a promising therapeutic modality in immunotherapy, with additional potential applications in regenerative medicine. Exosomes are now acknowledged for their significant roles in activating, suppressing, and monitoring immune pathways. Endogenous miRNAs can be functionally transferred via exosomes from donor cells to recipient cells, demonstrating their immunomodulatory effects [112,113]. They contribute to modulate immune responses in a different range of diseases, including cancer, cardiovascular conditions, nephropathy, and autoimmune disorders [114]. MSC-derived exosomes provide a cell-free alternate with comparable effects, easier management, no immunogenicity, and the ability to cross the blood-brain barrier [115]. On the other side, these exosomes exhibit anti-inflammatory properties and reduce immune activity similar to MSCs. Clinically, the immunomodulatory properties of MSC derived exosomes present a potential therapeutic strategy for SLE that is exerted by directing macrophages, T cells and Treg/TH2 cell. The production of autoantibodies in patients with SLE is contingent upon the activation of T-cell-assisted B cells. Consequently, modulating the activity of upstream T cells may help to limit excessive B-cell autoactivity in SLE [114]. Several invitro and *in vivo* studies were conducted to assess the effectiveness of simple or loaded/engineered MSC-derived exosomes on SLE pathology. Because the pathogenesis of lupus is caused by different factors, conventional drugs are only effective for a certain type of patients. Table 5 summarizes several studies that investigated the effects of MSC-derived exosomes from different sources on disorders related to SLE.

**Table 5:** Effect of MSC-Exo from different sources on disorders related to SLE.

Origin Cell	Study type	Cargo	Result	Reference
hUC-MSCs	*In vivo*	–	Inducing M2 Macrophage Polarization and Regulatory T Cell Expansion of MRL/lpr Mice Model	(124)
BM-MSC	*In vitro*	–	upregulation of CD206, B7H4, CD138, Arg-1, CCL20, and anti-inflammatory cytokines in MRL/lpr Mice Model	(119)
hUC-MSC-	*In vitro*	–	inhibited CD4^+^ T cells, increased the production of T helper (Th)17 cells, promoted the production of interleukin (IL)-17 and transforming growth factor beta1 (TGF-β1) of lupus mouse in vitro	(125)
hucMSCs	*In vitro*	–	promoted B cell apoptosis, prevented B cell over-activation, and reduced inflammation of patients B cells in vitro	(122)
hUC-MSCs	*In vitro*	tRNA-derived fragments (tRFs)	Significantly suppressed expression of M1 markers, and reduced the levels of TNF-α and IL-1β, increased M2 markers in macrophages	(120)
UC-BSCs	*In vitro*	miR-19b	UC-BSC- Exo treatment can target KLF13 expression by increasing the miR-19b level	(126)
hUC-MSCs	*In vivo*	microRNA-146a-5p	inhibit increased NOTCH1 expression, worsened bleeding and inflammation, and augmented M1 macrophage polarization while decreasing M2 macrophage polarization in lung tissues of DAH mice	(127)

Most current studies of potential therapeutic mechanisms using MSC exosomes loaded with specific miRNA and drugs provide great support for the development of new therapies for SLE. However, the effectiveness or side effects of transfecting several miRNAs or drugs together into exosomes are still unknown [116,117]. For instance, MSC-derived exosomes loaded with dexamethasone (Dex-MSC-EXOs) synergistically increased the anti-inflammatory inhibitory effect of CD4^+^ T cells compared to liposomal dexamethasone in lupus mouse model. Dex-MSC-EXOs induced cellular reprogramming by activating the glucocorticoid receptor (GR) signaling pathway, leading to an increase in the expression of anti-inflammatory factors and decrease in the expression of pro-inflammatory factors and cysteine-rich secretory protein LCCL-containing domain 2 (CRISPLD2) in CD4^+^ T cells [118].

*In vitro* treatment of macrophages isolated from the kidneys of MRL/lpr mice, as a SLE model, with exosomes derived from BM-MSCs resulted in the upregulation of CD206, B7H4, CD138, Arg-1, CCL20, and various anti-inflammatory cytokines, indicating that the macrophages were polarized toward a specific anti-inflammatory phenotype. These polarized macrophages exhibited low levels of reactive oxygen species (ROS) while demonstrating high efferocytosis activity and promoting the recruitment of Treg cells [119]. Transfer RNA–derived small RNA (tsRNA)-21109 that found in bone marrow mesenchymal stem cell (BM-MSC) derived exosomes inhibits the *in vitro* polarization of macrophages toward the M1 phenotype. This mechanism contributes to the identification of a new specific therapeutic target for SLE [120]. Gingival MSCs (GMSCs)- derived exosomes also exhibit both preventive and therapeutic effects on lupus, and their contents facilitated the M2 polarization of macrophages [111,121]. Furthermore, the coculture of exosomes derived from human umbilical cord mesenchymal stem cells (hucMSCs-Exo) with peripheral blood mononuclear cells (PBMCs) from SLE patients demonstrated an increase in the expression level of miR-155 in B lymphocytes, that measured by qRT-PCR. The miR-155 plays a significant regulatory role in B cell regulation, and this study also suggested that hucMSCs-Exo synergistically inhibit the autoreactivity of B cells [122]. Besides, UC-MSCs can release exosomes that promoted the anti-inflammatory polarization of macrophages, thereby decreased the SLE-associated diffuse alveolar hemorrhage in mouse models. Exosomes released by UC-MSCs significantly inhibit the production of pro-inflammatory cytokines, including IFN-γ, IL-2, and TNF-α, while simultaneously increasing the production of IL-10 as the anti-inflammatory cytokine [123].

The injection of BM-MSC-derived exosomes into a pristane-induced murine model of lupus nephritis facilitated the anti-inflammatory polarization of macrophages and increased the production of IL-17+ Treg cells. Notably, exosomes loaded with miR-16 and miR-21 downregulated PDCD4 and PTEN in macrophages, thereby enhancing the *in vivo* polarization of anti-inflammatory macrophage [119].

## 10 Limitations and future perspectives

While cell therapy holds promise for treating SLE, addressing some challenges is essential for its successful implementation and acceptance in clinical practice. The immune dysregulation in SLE patients is extremely complicated and varies among individuals; hence, patients may respond differently to the same therapy, complicating treatment protocols. On the other hand, manipulating the immune system can increase susceptibility of patients to infections. The mechanisms by which cell therapies exert their effects are not fully understood; so, there are few information about the efficacy of cell therapy in SLE patients. On the regards of cell manufacturing, processing the required cells can be technically challenging and costly. Lack of standardized protocols for manufacturing and administering cell therapies can hinder widespread adoption. Compliance with the regulatory landscape for approval of new therapies can be lengthy and complex. Ensuring consistent quality and potency of cell products is crucial for safety and efficacy. Among the cell therapy options, CAR T cell therapy offers potential benefits for treating SLE, but some challenges must be addressed such as identifying specific antigens, cytokine release syndrome (CRS), T cell exhaustion, the diversity of autoantigens in SLE, and risk of off-target effects. On the other side, Natural Killer (NK) CAR cell therapy offers several potential benefits for the treatment of SLE including lower incidence of cytokine release syndrome, transient controlled activation, direct cytotoxicity, and targeting wide range of abnormal cells [128,129]. Both CAR T-cell therapy and bispecific antibodies represent promising avenues for treating SLE, but they differ in their mechanisms, efficacy, and side effect profiles. While CAR T-cells may offer deeper and more sustained B-cell depletion, they also carry a higher risk of severe side effects. Bispecific antibodies offer a potentially flexible approach, easier dosage control, simplicity of application, reproducibility of results and scalability for mass production. CAR-T cell therapy requires a complex and time-consuming manufacturing process with need for lymphodepletion prior to the infusion [130]. Further research is needed to determine the optimal use of these therapies in different SLE patient populations and to refine treatment strategies to maximize benefits while minimizing risks. The application of exosomes as cell free approach for SLE diagnosis and treatment was investigated and showed some benefits. Exosomes can be engineered to deliver therapeutic agents (like drugs or RNA) directly to target cells, minimizing side effects and enhancing efficacy. They can play a role in modulating immune responses, potentially correcting the dysregulation in the SLE. However, significant challenges about the therapeutic application of exosomes like purification with high quality and quantity, lack of standardized protocols for exosome characterization and analysis, the heterogeneous nature, and targeting specificity must be solved to improve the efficiency exosome therapy for SLE. Future research is essential to fully understand its efficacy and optimize treatment protocols for SLE patients.

## 11 Conclusion

SLE remains a complex autoimmune disorder with substantial unmet clinical needs, particularly in patients who are unresponsive to conventional therapies. The chronic nature of the disease, accompanied by relapses, organ damage, and treatment-associated toxicity, emphasizes the urgency for alternative therapeutic strategies. Over recent years, innovative cell-based therapies have emerged as promising tools in the management of refractory SLE. HSCT, MSCs, CAR T cells, iPSCs, and MSC-derived exosomes have all demonstrated encouraging preclinical and clinical results by modulating immune responses, reducing autoimmunity, and promoting tissue regeneration. Each therapeutic modality brings unique advantages and limitations. HSCT can potentially “reset” the immune system but carries risks of relapse and infection. MSCs, particularly from allogeneic sources, offer immunomodulatory benefits with lower immunogenicity, while CAR T cell therapy provides antigen-specific targeting but requires further refinement for broader applicability. iPSCs and exosome-based therapies, though in earlier stages, present exciting avenues for personalized and cell-free treatment approaches. Nonetheless, significant challenges remain, including heterogeneity in patient response, manufacturing complexity, long-term safety, and the need for standardized protocols. Future research should focus on optimizing treatment protocols, enhancing targeting specificity, and conducting large-scale, controlled clinical trials to validate the long-term efficacy and safety of these novel therapies. Ultimately, the integration of advanced cell-based strategies may revolutionize the treatment landscape of SLE, moving closer toward achieving sustained remission and improved quality of life for patients.

## CRediT authorship contribution statement

**Zeinab Zarei-Behjani:** Writing – review & editing, Writing – original draft, Validation, Supervision, Investigation, Conceptualization. **Arghavan Hosseinpouri:** Writing – original draft, Investigation. **Maryam Fotoohi:** Writing – original draft. **Akram Shafiee:** Writing – original draft. **Dorna Asadi:** Writing – original draft.

## Ethics approval and consent to participate

Not applicable.

## Consent for publication

Not applicable.

## Availability of data and materials

Not applicable.

## Funding

Not applicable.

## Declaration of competing interest

The authors declare that they have no known competing financial interests or personal relationships that could have appeared to influence the work reported in this paper.

## Data Availability

No data was used for the research described in the article.
